# Hospital-Based Back Surgery: Geospatial-Temporal, Explanatory, and Predictive Models

**DOI:** 10.2196/14609

**Published:** 2019-10-29

**Authors:** Lawrence Fulton, Clemens Scott Kruse

**Affiliations:** 1 Department of Health Administration Texas State University San Marcos United States

**Keywords:** back surgery, neurosurgeon, elastic net, lasso, ridge, random forest, geospatial mapping, health economics, obesity, practice variation

## Abstract

**Background:**

Hospital-based back surgery in the United States increased by 60% from January 2012 to December 2017, yet the supply of neurosurgeons remained relatively constant. During this time, adult obesity grew by 5%.

**Objective:**

This study aimed to evaluate the demand and associated costs for hospital-based back surgery by geolocation over time to evaluate provider practice variation. The study then leveraged hierarchical time series to generate tight demand forecasts on an unobserved test set. Finally, explanatory financial, technical, workload, geographical, and temporal factors as well as state-level obesity rates were investigated as predictors for the demand for hospital-based back surgery.

**Methods:**

Hospital data from January 2012 to December 2017 were used to generate geospatial-temporal maps and a video of the Current Procedural Terminology codes beginning with the digit 63 claims. Hierarchical time series modeling provided forecasts for each state, the census regions, and the nation for an unobserved test set and then again for the out-years of 2018 and 2019. Stepwise regression, lasso regression, ridge regression, elastic net, and gradient-boosted random forests were built on a training set and evaluated on a test set to evaluate variables important to explaining the demand for hospital-based back surgery.

**Results:**

Widespread, unexplained practice variation over time was seen using geographical information systems (GIS) multimedia mapping. Hierarchical time series provided accurate forecasts on a blind dataset and suggested a 6.52% (from 497,325 procedures in 2017 to 529,777 in 2018) growth of hospital-based back surgery in 2018 (529,777 and up to 13.00% by 2019 [from 497,325 procedures in 2017 to 563,023 procedures in 2019]). The increase in payments by 2019 are estimated to be US $323.9 million. Extreme gradient-boosted random forests beat constrained and unconstrained regression models on a 20% unobserved test set and suggested that obesity is one of the most important factors in explaining the increase in demand for hospital-based back surgery.

**Conclusions:**

Practice variation and obesity are factors to consider when estimating demand for hospital-based back surgery. Federal, state, and local planners should evaluate demand-side and supply-side interventions for this emerging problem.

## Introduction

### Background

In 2012, there were 3689 practicing board-certified neurosurgeons in the United States [1]. That number was largely unchanged in 2016 [2]. During these years, demand for back surgery (Current Procedural Terminology [CPT] codes beginning with the digit 63) increased by 49% from 311,028 to 464,391, and by the end of 2017, that increase was 60% [3]. CPT *63* medical codes are a series of spinal procedures including laminectomies, laminotomies, decompressions, and corpectomies. These procedures do not include needle decompression, catheter implantation, and, as of 2019, endoscopic decompression [4]. Given the stable supply and increasing demand, it is not surprising that the average payment procedure increased from US $4166 to US $4859 from 2012 to 2016 and to US $5452 by the middle of 2018, an effective 4.5% inflation rate [3]. Forecasting models that address increasing demand are necessary to evaluate potential supply and demand-side interventions.

Unsurprisingly, there is a marked variation in the treatment of back disorders such as spondylolisthesis [5]. This variation affects costs [6] as well as outcomes [5] associated with back surgery. The implication of this variation is increased demand. By evaluating the current geographic demand, policy makers can prioritize efforts for cost and variation reduction by evaluating those states and counties that exhibit high practice area variation, implementing evidence-based best practice policies and guidelines, educating populations about obesity risks, and implementing interventions for those at risk of obesity (eg, those living in food deserts).

During the same time that back surgeries have increased, adult obesity rates in the United States have also increased. The rate of this increase was 5% from 2012 to 2016 (34.9%-39.6%) [7]. Obesity has been linked to increased costs of medical care [8]. Although obese patients benefit from at least some back surgeries, they do not fare as well as nonobese patients [9]. Although obesity has been linked to back pain [10], no studies were found that directly link obesity to back surgery requirements. This study evaluated that relationship as well.

### Objectives

This study addressed 3 specific aspects of hospital-based CPT 63 surgery. First, a geospatial-temporal analysis by zip code is conducted to describe the previous and current demand for CPT 63 surgery. The significance of this geospatial-temporal analysis is that practice variation is highlighted for evaluation by federal, state, and local policy makers. Second, forecasting models estimate the demand and payments overall, by census region and by state. These models are also designed for state policy makers to assess potential supply- and demand-side intervention requirements. Third, explanatory models are developed to correlate obesity rates and financial, technical, workload, temporal, and geospatial variables with demand for CPT 63 procedures. This analysis does not appear to be previously investigated and is an important but overlooked correlational analysis. The study focused specifically on hospital-based knee surgery with CPT 63 codes (some of which reflect inpatient procedures) and was delimited to knee surgery only.

## Methods

### Data

Definitive Healthcare provided the hospital, zip code, and state-level procedure and cost data from January 2012 to June 2018 through the hospital *revenue center analytics* query, which includes queries by CPT code. Data in Definitive Healthcare are derived from the Standard Analytical Files by the Centers for Medicare and Medicaid Services (CMS)]. From these data, the organization uses undisclosed algorithms to estimate all-payor claims. Columns with fewer than 11 claims or procedures are not shown because of privacy requirements [3]. For this analysis, only complete annual data from 2012 to 2017 were used, as the CMS datafile and thus the associated estimates from January 2018 through June 2018 were approximately only 93% complete [3].

The Centers for Disease Control and Prevention’s Behavioral Risk Factor Surveillance System (BRFSS) prevalence data provided the information for state-level adult obesity rates by year, from 2012 to 2017 [11]. Guam, Puerto Rico, and the US Virgin Islands were excluded from the analysis because of small sample sizes in both datasets.

### Geospatial Analysis

Heat maps are used to plot the zip code unit of analysis procedure data by year. Heat maps provide the intensity of the number of claims by time and geographic region. These types of maps have been used for improving minority health surveillance [12], examining birth outcomes [13], and evaluating a variety of other applications in health care. The value in geospatial-temporal analysis is the graphical depiction of change in demand over time. A video display from 2012 to 2017 with standardized heat intensities provides an animated view of the change in demand by location. An analysis of cost and demand centers is then provided.

### Forecasting Analysis

The data in the Definitive Healthcare dataset are nonseasonal as they provide annual-level observations by the hospital unit of analysis. Even so, generating nonseasonal forecasting models that have predictive capability on a blind withhold set at the proper level of aggregation can provide decision support for supply- and demand-side interventions. These types of models have found support in many areas of health care such as radiology [14] and Alzheimer disease [15].

To this end, hierarchical time series (HTS) [16] using R statistical software [17] evaluated the number of claims as a function of time series components. An HTS recognizes that data are aggregated at various levels. In this case, the hierarchy evaluated include the states, the census regions, and the nation. The models are built on a training set of data for the years 2012 to 2015 and forecast on a blind test set, years 2016 and 2017. Although Bayesian hierarchical models have been used for spatially correlated health outcomes and utilization rates [18], there is no readily found use of HTS for prediction in health care.

To understand HTS, one needs to only consider a single medical system that operates in 2 separate states with 3 hospitals per state. There are then 4 basic ways using which one might forecast annual visits as an example:

1. A forecast might be generated for each hospital, aggregated at the state level and then further aggregated at the system level. A variety of different forecast methods might be used to generate the forecasts. The term for this method is bottoms up.
2. Forecasts might be generated at the state level and then disaggregated (eg, via historical proportions) to the hospitals and aggregated to the system level. Again, the forecasts might be generated in multiple ways. The term for this method is middle out.
3. Forecasts might be generated at the system level (via multiple methods) and then disaggregated to all levels below (eg, proportions). The term for this is top down.
4. One might take some combination of the previous methods to minimize forecast error. This is an ensemble method that might be termed the optimal reconciliation approach, which is optimal if the forecasts are unbiased [19].

To avoid selection bias, all methods were evaluated for performance on the test set. Furthermore, the method for forecasting at these levels of hierarchy was using autoregressive integrated moving average (ARIMA) components and as well as smoothed error and trend components (exponential, trend, seasonality [ETS] without seasonality).

ARIMA models focus on autocorrelation of components for stationary time series data. The *AR* components are autoregressive terms, offset by time. For example, the number of claims at time *t* might be forecast by using the number of claims at time *t-1*. This would be an AR1 model, as there is 1 offset. As ARIMA models assume stationarity of the time series for forecasting, 2 other components are necessary. The first is differencing or *integration*, the *I* in ARIMA, which helps stabilize the mean (whereas transformations help stabilize the variance). Although seasonality and trend might make an ARIMA nonstationary, differencing often corrects this. Sometimes, more than 1 difference is required to make the time series stationary, for example, *y_t-_y_t-1_-(y_t-1_-y_t-2_*) is a 2d order differencing. The last component, the *MA* or moving average, corrects for autocorrelated errors as well. This component averages previous observation(s) with the previous forecast(s) [19].

ETS models have 3 components: error, trend, and seasonality. As the data in this study are not seasonal, only the error smoothing (identical to a moving average) and the trend component (a Holt model [20]) are evaluated.

With HTS bottom-up models, a separate ARIMA/ETS is built for each bottom-level component. For middle-out models, all middle-level components have separate forecasts. For top-down models, a single forecast is generated and proportioned down to the lower levels.

### Explanatory Analysis

Stepwise linear regression (both forward and backward), lasso regression, robust regression, elastic net regression, and extreme gradient-boosted random forests are built on unaggregated data as well as state-level aggregated data to estimate the number of claims. These models are built on a random 80% training set (10,771 unaggregated, 245 aggregated observations) and evaluated on a 20% withhold set (2693 unaggregated, 61 aggregated) as well. The total number of valid observations were 13,464 unaggregated and 306 aggregated. The primary hypothesis is that the inclusion of obesity rates as an independent variable will yield better explanatory models for both the number of claims and the payment per claim.

Stepwise linear regression based on minimum Akaike Information Criterion was selected over best subset because of the computational complexity. By using forward and backward simultaneously, variables are added in sequence but might be removed if they no longer contribute to the model [21].

**Figure 1:**
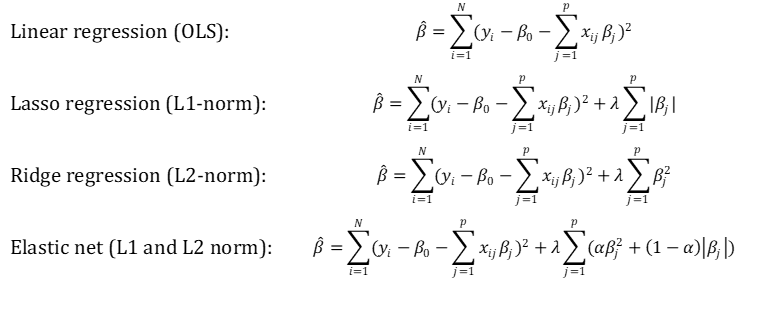
Argmin equations for the regression models.

Lasso regression is a form of constrained regression that penalizes a model that selects too many variables by using an L1-norm formulation (absolute value), whereas ridge regression is similar but penalizes using an L2-norm formulation (squared coefficient estimates). Elastic net uses a weighted L1 and L2 norm penalty function to reduce the number of coefficients in the model. Formulae for estimating the parameters of the linear model, the lasso regression, the ridge regression, and the elastic net are shown in Figure 1.

**Figure 2:**
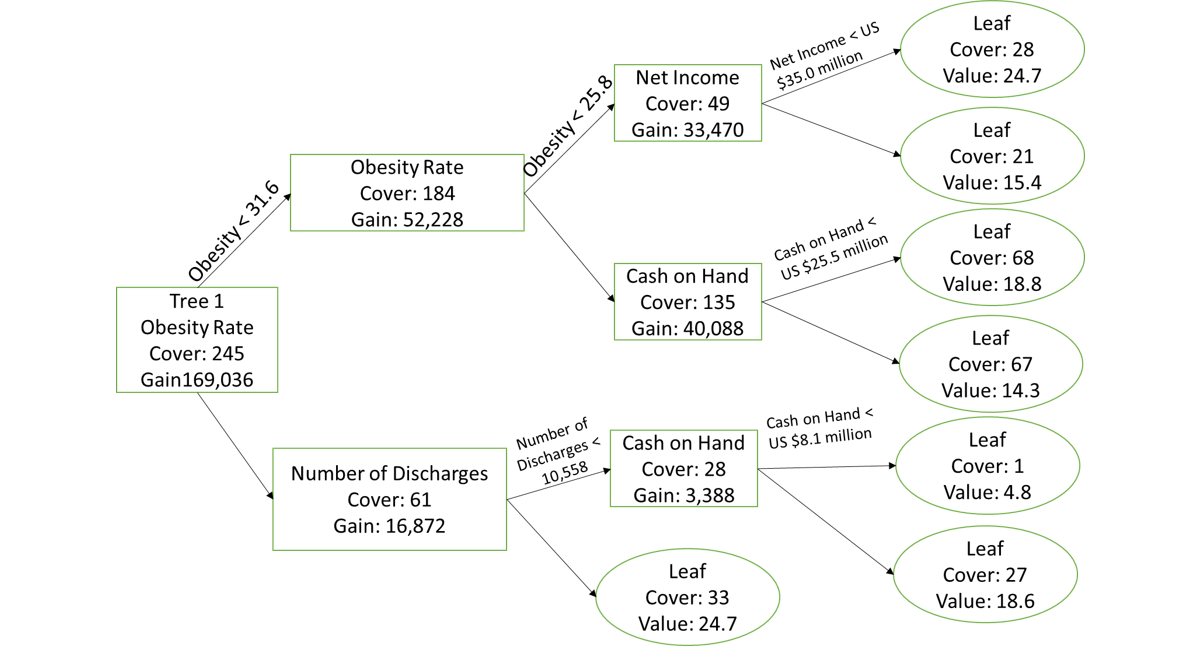
An example of a single tree model with 3 branches. The graph was produced by the xgboost package of R. (NumDischarges indicates the number of discharges).

Random forests, a machine learning technique, use an ensemble of decorrelated tree models and average the estimates of those trees to build forecasts. A tree model itself classifies counts of observations by splitting variables at points based on some decision criteria. An example of a tree with a depth of 3 (3 branches) is given in Figure 2, which splits observations by obesity rate less than 31.63 and then again by obesity rate less than 25.75 and number of discharges less than 10,558.78 and then finally by net income less than US $35,018,392, cash less than US $25,522,424, and cash less than US $8,122,498 [21]. The graph was produced by the xgboost package of R [22]. Gradient-boosted random forests optimize a cost function based on the (pseudo-)residuals of a function using nonlinear optimization techniques. Essentially, the residuals of each tree in the forest are refitted with the possible independent variables in another tree model to estimate a better fit of the original function. Often, a learning rate (shrinkage) is applied to the residuals to allow for better generalization. A discussion of gradient boosting is available in Chapter 10 of *Elements of Statistical Learning* [21].

### Variables

All the considered variables from the Definitive Healthcare dataset are shown in Table 1 with reasons for exclusion/inclusion. Most variables were, by default, included during analysis; however, those variables that were linear combinations of each other or were necessarily unknowable when forecasting CPT 63 codes were omitted.

There is 1 primary dependent variable of interest: number of claims for CPT 63 codes. This variable is measured at the hospital level over time and is also aggregated by zip code/year for geospatial mapping and by state/year for forecasting and additional modeling analysis. The number of claims include third-party invoices provided by the hospital, regardless of the payer. The number of claims provides a measure of the met demand for services.

For the geospatial and temporal analyses, the variables year and zip code (aggregated hospital-level data) are used to describe the intensity of both the number of claims and the payment per claim. Zip code provides a high resolution for geographic claims data. For the HTS forecasting analysis, time components are used without external regressors.

Explanatory stepwise regression, lasso regression, ridge regression, elastic net, and gradient-boosted random forest models investigate financial variables, technical variables, workload variables, geospatial variables, a temporal variable (year), and obesity rates (defined as the proportion of individuals with a body mass index greater than or equal to 30%). A discussion of each of the variable groups and variables follows.

The financial variables investigated include net patient revenue, net income, cash on hand, total assets, total liabilities, and proportion of Medicare/Medicaid reimbursement. The financial variables were carefully selected from the set of available financials such that they are not a linear combination of other variables or nearly a linear combination (see Table 1). Although available, total payments and charges for CPT 63 were not used in the models, as they (1) would not be known in advance and (2) would necessarily be direct functions of the number of claims.

Quantitative workload variables include the number of staffed beds, discharges, emergency room visits, surgeries, affiliated physicians, and employees. Categorical technical variables include ownership type, medical school affiliation, and hospital type. These variables are investigated because of their availability and possible confounding effects. Geographic variables include urban/rural location, state, and zip code. These variables are important in evaluating practice area variation and associated effects.

Obesity rates are of interest to the study. These rates are assigned based on the state, as county and zip code level data are not available. This independent variable is of importance to the study.

**Table 1:** Variables in the study.

Variable	Type	Definition	Scale of measurement
Number of claims	Dependent	Filed third-party claims	{0, 1, 2, ...k}
Obesity rate	Obesity	Percentage obese by state	[0%, 40%]
CPT^a^ 63 payments	Financial	Total CPT 63 payments	US $
CPT 63 charges	Financial	Total CPT 63 charges	US $
Net patient revenue	Financial	Total revenue from patients	US $
Total revenues	Financial	All revenue, patient-related or otherwise	US $
Net income	Financial	Revenues less expenses	US $
Total expenses	Financial	Total dollars attributed to expenses	US $
Cash on hand	Financial	Funds immediately available	US $
Total assets	Financial	Current and noncurrent assets	US $
Total liabilities	Financial	Current and long-term debt	US $
Percentage Medicare/Medicaid	Financial	Percentage claims from either source	%
State	Geospatial	Hospital's state (address)	AK, AL, ...
Zip code	Geospatial	5-digit hospital zip code	78666, ...
Geographic classification	Technical	Rural or urban location	Rural, urban
Ownership	Technical	Hospital ownership	Nonprofit, profit, government
Medical school affiliation	Technical	Level of affiliation if any	Graduate, major, limited, none
Hospital type	Technical	Type of hospital	Short-term acute, children’s, etc
Year	Temporal	Year of report	2012, 2018
Number of staffed beds	Workload	Per Medicare report	{0, 1, ...n}
Number of discharges	Workload	Total number of inpatient discharges	{0, 1, ...n}
Number of Medicare discharges	Workload	Number of Medicare discharges	{0, 1, ...n}
Estimated number of emergency room visits	Workload	Number of emergency room visits	{0, 1, ...n}
Total surgeries	Workload	Number of surgeries	{0, 1, ...n}
Total acute days	Workload	Number of acute bed days	{0, 1, ...n}
Number of affiliated physicians	Workload	Number of affiliated physicians	{0, 1, ...n}
Number of employees	Workload	Number of employees	{0, 1, ...n}

^a^CPT: Current Procedural Terminology.

## Results

### Descriptive Statistics: Missing Data

Missing data were present in the Definitive Healthcare dataset. As the percentage of missing data was small, the data were imputed via regression trees (simple imputation). The total number of valid observations at the hospital unit of analysis from January 2012 to December 2017 was 13,769. There were 2244 unique zip codes with data resulting in 13,464 observations from 2012 to 2017, although many of these were true zeros. Aggregated at the state level, there were 306 observations of the 50 states plus the District of Columbia over the 6-year span.

### Descriptive Statistics: Quantitative Data

Important descriptive statistics for the Definitive Healthcare data are shown (Table 2). The average number of CPT 63 claims by hospital by year was 182, and the average payment was over US $4045.99, about 50.37% of mean charges (US $8,032.13). On average, hospitals performing these claims were large (227 beds with 1629 employees and 299 affiliated physicians). These hospitals had on average positive net income (US $22 million) and assets exceeding liabilities. Overall, 45% of their patients used Medicare or Medicaid reimbursement.

**Table 2:** Descriptive statistics for all years and all hospitals (N=13,769). The “K” suffix indicates dollars in thousands, while the “M” suffix indicates dollars in millions.

Variable	Mean (SD)	Median	Minimum	Maximum
Claims, n	182 (244)	89	11	3592
Payments/claim, US $	4045.99 (2448.96)	3659.12	0	34,975.76
Payments, US $	767.9K (1097.8K)	366.2K	0	18,966.3K
Charges, US $	1517.6K (2467.4K)	616.4K	0	35,317.2K
Charges/claim, US $	8032.13 (6993.90)	6370.08	0	137,058.80
Net patient revenue, US $	343.8M (430.4M)	217.5M	−98.6M	5340.9M
Net income, US $	22.8M (102.8M)	11.3M	−1648M	1316.0M
Cash, US $	30.3M (145.3M)	2.8M	−1992.7M	3597.8M
Total assets, US $	443.0M (820.1M)	203.5M	−231.7M	9969.4M
Total liabilities, US $	178.2M (465.7M)	69.2M	−2583.8M	6372.4M
Staffed beds, n	227.45 (201.36)	177.00	1.00	2626
Discharges, n	11,822.61 (11,395.52)	8899.00	1.00	127,600
Emergency room visits, n	47,439.59 (39,580.09)	39,209.00	0	543,457
Surgeries, n	9643.21 (9666.56)	7019.00	0	134,638
Affiliated physicians, n	298.57 (333.43)	198.00	1.00	3483
Employees, n	1629.46 (2044.45)	1027.00	7.00	24,673
Percentage of Medicare/Medicaid, %	0.45 (0.14)	0.44	0	1
Obesity rate, n	29.44 (3.42)	29.92	20.20	38

The number of hospitals reporting CPT 63 claims increased by 91 from 2012 to 2017. Charges increased from US $2.115 million to US $4.75 million, whereas payments increased from US $1.233 million to US $2.467 million. The proportion of charges paid fluctuated between 45% and 58%. The number of claims increased from 320K to 504K, a 60% increase (Table 3).

Variation across states from 2012 to 2017 for CPT 63 is impressive. The maximum average payment per claim was in Delaware (US $5190.62); however, the number of actual claims was small (5569). New York had the second highest payment per claim (US $5043.79) with 72,186 claims. Texas had the largest number of claims (260,208), yet the average payment per claim was only US $4223.22. On average, 60% of charges were paid (Table 4).

Obesity rates by state have increased from 2012 to 2017 (Table 5). In 2012, the mean obesity rate per state was 27.95%. By 2017, this rate was 30.59%; however, this increase is not weighted by population size. As discussed previously, the aggregate increase for the United States from 2012 to 2017 was 5% (34.9% to 39.6%) [7]. The state data include the District of Columbia (51 observations per year) but are not population weighted.

**Table 3:** Average statistics by year show the growth in both claims and payments.

Year	Hospitals, n	Total payments, in millions of US $	Total charges, in millions of US $	Total claims, n	Payments/claim, in US $	Charges/claim, in US $
2012	2248	1232.61	2114.56	320,371	3847	6600
2013	2293	1460.80	2698.34	372,155	3925	7251
2014	2306	1517.88	3396.62	410,317	3699	8278
2015	2290	1747.68	3688.72	428,813	4076	8602
2016	2336	2147.48	4247.37	472,004	4550	8999
2017	2339	2466.92	4750.68	504,626	4889	9414

**Table 4:** Payments, charges, number of claims, payment per claim, charge per claim, and percentage of charges paid by state.

State	Payments, in millions of US $	Charges, in millions of US $	Number of claims, n	Payment/claim, in US $	Charge/claim, in US $	Percentage paid, %
Alaska	21.17	37.94	4387	4826.27	8649.31	55.80
Alabama	232.76	419.16	75,486	3083.47	5552.77	55.50
Arkansas	124.27	200.21	33,087	3755.85	6051.07	62.10
Arizona	175.60	526.67	41,274	4254.39	12,760.42	33.30
California	563.57	1748.02	113,410	4969.31	15,413.29	32.20
Colorado	147.28	440.24	41,537	3545.72	10,598.65	33.50
Connecticut	72.96	139.43	17,179	4247.17	8116.50	52.30
District of Columbia	26.31	53.90	5655	4651.78	9531.16	48.80
Delaware	28.91	25.94	5569	5190.62	4657.34	111.50
Florida	546.44	1543.23	131,442	4157.29	11,740.79	35.40
Georgia	318.64	654.84	79,401	4013.02	8247.29	48.70
Hawaii	12.02	22.03	2802	4291.08	7861.44	54.60
Iowa	131.47	277.09	34,419	3819.62	8050.58	47.40
Idaho	93.02	163.59	23,514	3956.12	6957.22	56.90
Illinois	361.17	706.39	81,960	4406.60	8618.73	51.10
Indiana	361.28	800.98	87,204	4142.93	9185.09	45.10
Kansas	193.40	310.85	39,992	4836.07	7772.78	62.20
Kentucky	215.53	306.93	50,147	4298.00	6120.69	70.20
Louisiana	204.21	423.83	55,726	3664.61	7605.54	48.20
Massachusetts	210.91	267.24	42,609	4950.01	6272.03	78.90
Maryland	83.04	96.72	34,732	2390.89	2784.66	85.90
Maine	69.88	78.25	21,468	3255.23	3645.18	89.30
Michigan	311.32	389.73	70,173	4436.41	5553.81	79.90
Minnesota	191.90	279.98	47,410	4047.73	5905.41	68.50
Missouri	362.53	487.40	83,085	4363.33	5866.30	74.40
Mississippi	159.04	400.04	39,059	4071.72	10,241.97	39.80
Montana	54.19	86.78	14,106	3841.66	6151.89	62.40
North Carolina	503.97	886.27	126,344	3988.87	7014.74	56.90
North Dakota	36.87	43.36	9081	4060.13	4774.58	85.00
Nebraska	94.49	184.44	24,310	3,886.98	7586.98	51.20
New Hampshire	74.81	120.38	19,091	3918.49	6305.40	62.10
New Jersey	169.55	328.00	35,479	4778.93	9244.90	51.70
New Mexico	31.17	68.52	8583	3631.59	7983.19	45.50
Nevada	97.33	190.03	24,617	3953.67	7719.64	51.20
New York	364.09	395.74	72,186	5043.79	5482.21	92.00
Ohio	396.47	743.30	87,427	4534.88	8501.92	53.30
Oklahoma	239.62	494.62	51,639	4640.38	9578.46	48.40
Oregon	180.39	280.10	38,328	4706.54	7307.94	64.40
Pennsylvania	383.17	736.78	85,334	4490.29	8634.05	52.00
Rhode Island	15.94	16.37	3772	4225.09	4339.10	97.40
South Carolina	293.49	492.03	60,977	4813.14	8069.03	59.60
South Dakota	70.42	204.78	17,870	3940.84	11,459.54	34.40
Tennessee	289.03	619.13	79,948	3615.26	7744.18	46.70
Texas	1,098.91	2,450.47	260,208	4223.22	9417.34	44.80
Utah	143.63	138.51	31,168	4608.37	4443.86	103.70
Virginia	231.88	466.58	56,436	4108.69	8267.36	49.70
Vermont	12.44	24.56	3578	3476.00	6864.49	50.60
Washington	264.29	669.87	66,457	3976.84	10,079.74	39.50
Wisconsin	161.13	283.08	39,182	4112.30	7224.71	56.90
West Virginia	117.51	131.28	23,852	4926.48	5504.07	89.50
Wyoming	29.94	40.79	6149	4868.89	6633.27	73.40

**Table 5:** State statistics for the proportion of the population identified as obese by the Behavioral Risk Factor Surveillance System by year.

Statistic	Year					
	2012	2013	2014	2015	2016	2017
Mean (SD) proportions	27.95 (3.38)	28.65 (3.44)	29.23 (3.42)	29.28 (3.87)	29.78 (3.74)	30.59 (3.86)
Median proportions	27.60	29.40	29.60	29.83	29.92	31.30
Range	14.2	13.8	14.6	16.0	15.39	15.42
Minimum	20.5	21.3	21.3	20.2	22.27	22.64
Maximum	34.7	35.1	35.9	36.2	37.66	38.06
Count	51	51	51	51	51	51

### Descriptive Statistics: Categorical Data

Of the 13,769 hospital observations, 3153 were rural and the remaining 10,616 were urban. Most hospital observations were classified as voluntary nonprofits (8866, 64%), whereas proprietary corporations and government entities constituted 3426 (25%) and 1466 (11%), respectively, (11 hospital observations had no ownership specification). Most of the hospital observations (8311, 60%) had no affiliation with medical schools. The vast majority of the observations were from short-term acute care hospitals (13,040, 95%) with nearly all of the remainder (678, 5%) associated with critical access hospitals.

### Descriptive Statistics: Correlational Analysis

**Figure 3:**
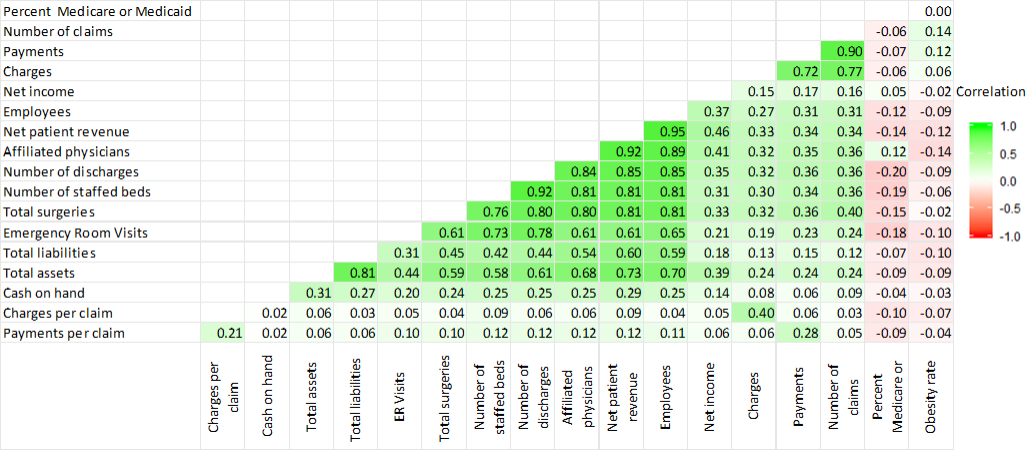
The correlation matrix depicts the variable relationships. The X’s indicate no statistically significant correlation. Owing to the large sample size, nearly all correlations are statistically significant at the alpha=.05 level. The matrix was produced using ggcorrplot.

Hierarchical clustered correlational analysis revealed strong relationships among many of the quantitative variables. Payments and claims are (as to be expected) highly correlated (*r*=0.9). Most financial and workload metrics are highly correlated as well (eg, net patient revenue and the number of employees; *r*=0.95). Owing to the large sample size, nearly all correlations are statistically significant at the alpha=.05 level (see Figure 3). The matrix was produced using ggcorrplot [23].

**Figure 4:**
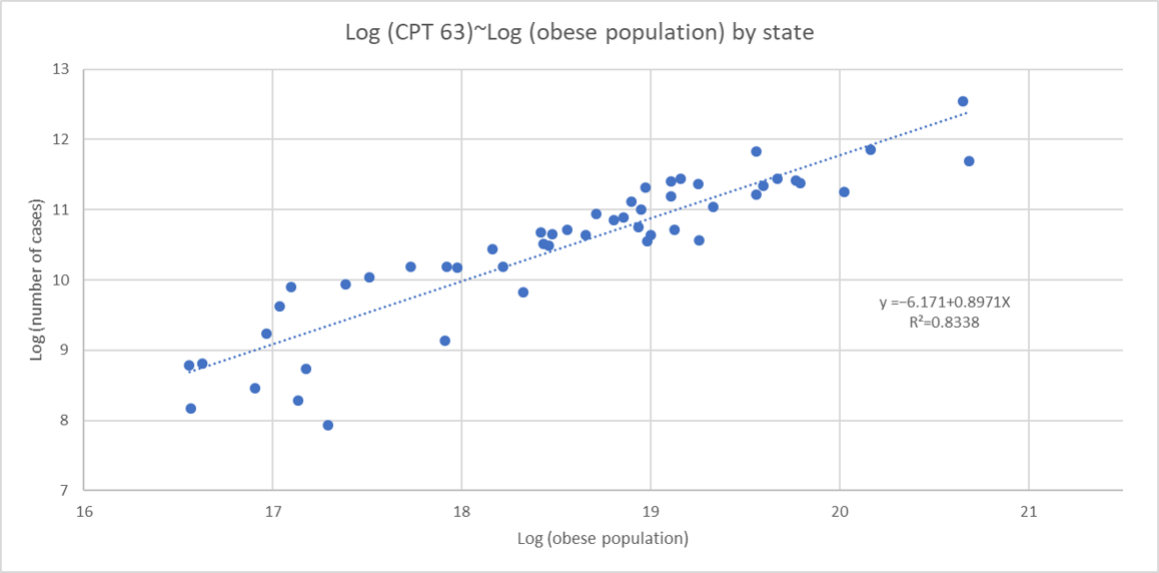
Correlation between the natural logarithm of obesity rates and the natural logarithm of the number of CPT 63 surgeries performed by hospitals.

The inclusion of obesity in this study is because of a correlational finding that the number of CPT 63 procedures appears to be influenced by state obesity rates at the aggregate level (Figure 4 [24]). The question, though, is whether this apparent correlation in the logs is sustained when other financial, geographic, technical, and temporal variables are considered.

### Exploratory Data Analysis: Feature Engineering and Transformations

Although random forest regression models are scale invariant, traditional regression techniques such as stepwise, lasso, ridge, and elastic net are not [21]. Investigating transformations to achieve multivariate normality (assuming random-effects regression) is important to meet model assumptions. Furthermore, time series forecasting often benefits from these same techniques [16]. In addition, investigating additional features that might be generated from the existing ones through linear combinations and other methods often results in disentangling collinear variables and finding interesting relationships that might otherwise remain undiscovered.

A multivariate Box-Cox transformation was run using the *car* package in R [25] for all modeled quantitative variables simultaneously after these variables had been location adjusted to make the variables strictly positive, definite, and scale adjusted by dividing by the standard deviation. Multivariate Box-Cox seeks to find power transformations (values of λ for each variable) that make the data multivariate normal enough for use in traditional linear models [26]. These transformations help alleviate the problem of collinearity and address multivariate normal assumptions of random-effects regression. The null hypothesis is that the proposed transformation generated through the transformation is a good fit. The alternative is that it is not a good fit. The proposed transformation was a vector of primarily natural logarithms (values near zero) with some exceptions. The likelihood ratio test resulted in a *P* value >.99, indicating that the assumption of multivariate normality cannot be rejected. The actual vector of transformations follows: λ={0.1, 0.3, 0.33, 0.38, 0.21, −0.07, 1.66, 0.76, −0.03, 0.55, 0.28, 0.17, 1.03, 1.04} for x={number of claims, number of staffed beds, number of discharges, ER visits, total surgeries, net patient revenue, net income, cash, total assets, total liabilities, affiliated physicians, employees, percent Medicare/Medicaid, obesity rate}, respectively.

**Figure 5:**
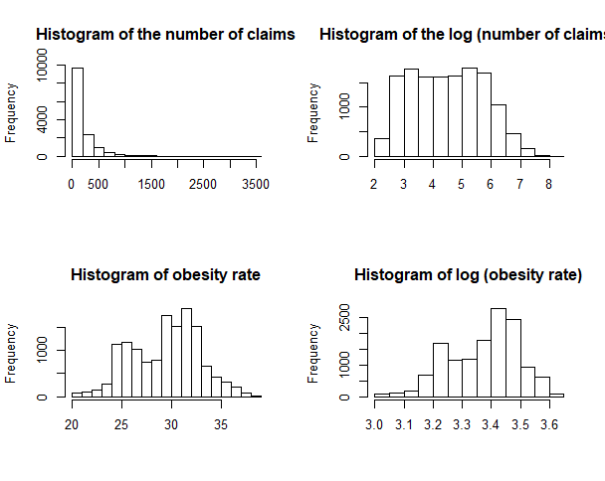
The untransformed and transformed histograms of the number of claims and the obesity rate variables.

Univariate histograms for the number of claims and obesity pre- and posttransformation are in Figure 5. The transformed graph of the number of claims shows some slight skew but is otherwise unremarkable. However, the graph of obesity rates is telling. Although the transformation fails to reject the assumption of multivariate normality, the obesity graph is bimodal. It is possible that linear-in-parameter models will not be able to correctly fit the importance of this variable, whereas tree-based models will find patterns.

### Geospatial Analysis Results: Zip Code Unit of Analysis

**Figure 6:**
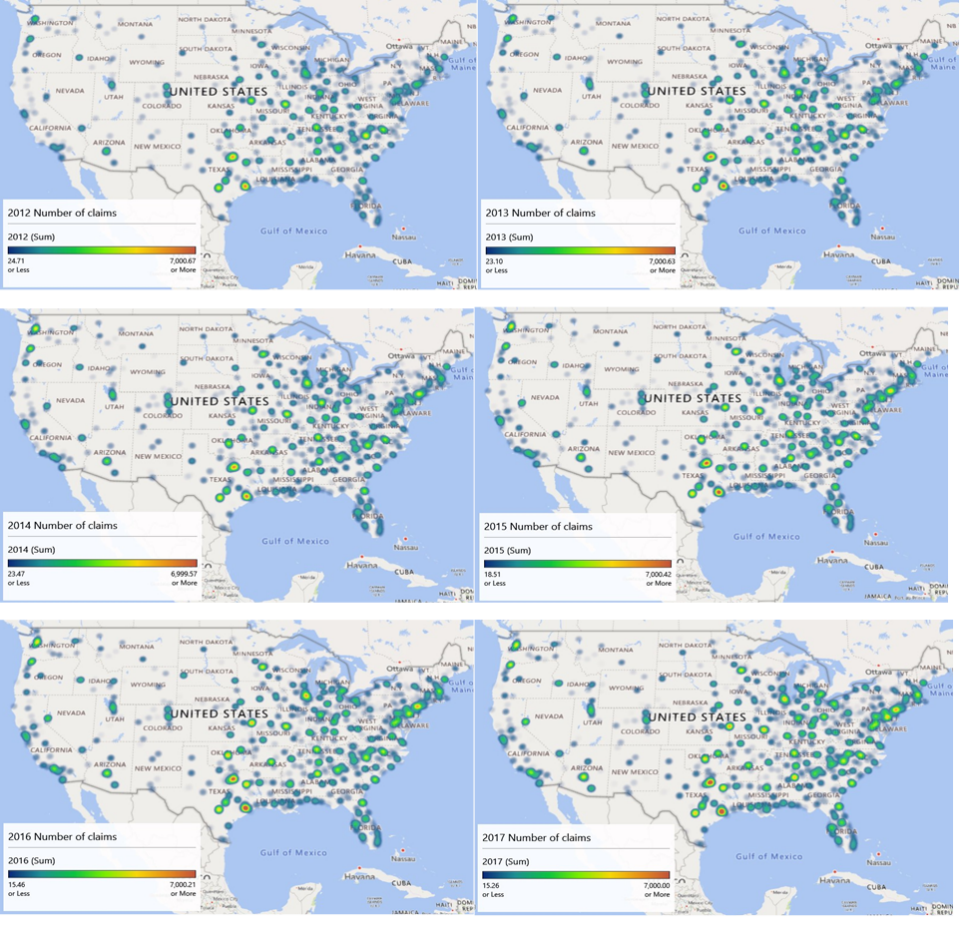
Geospatial analysis of all CPT 63 claims from 2012 through 2017.

Geospatial heat map analysis of CPT 63 number of claims by year and parsed by zip code is shown in panels (Figure 6). The maximum scale is approximately 7000 claims for each diagram to allow for comparison across years. Multimedia Appendix 1 shows this analysis in video format.

In 2012, there was very little high-intensity activity (Houston and Dallas, Texas, primarily, with some activity in the Carolinas). The Eastern seaboard has activity, but it is not intense, and the Western seaboard has minimal activity, except near Seattle.

By 2013, the Eastern seaboard (particularly New Jersey) has increased in intensity, and the areas around Chicago and Salem, Oregon, are emerging as well. Houston and Dallas remain the most intense regions for the number of claims.

In 2014, the number of claims in Seattle and San Antonio, Texas, shifted these cities to high-intensity areas (some red visible). It must be noted that 3 of the 4 cities with visible red tint are in Texas (San Antonio, Dallas, and Houston).

The year 2015 saw increasing intensity in both the New Jersey area and Chicago, Illinois. These 2 areas joined Houston, Dallas, San Antonio, and Seattle as high-intensity claims areas. Despite their populations, neither California nor Florida experienced the claims intensity of Texas.

Houston, Dallas, San Antonio, Seattle, Longview (Texas), Oklahoma City, the New Jersey area, and St. Louis were the notable areas of high intensity in 2016. The California coast became more intense along with Salt Lake City.

By 2017, the Eastern seaboard intensified (New Jersey, Delaware, Pennsylvania, Virginia, and District of Columbia) along with Phoenix, Arizona, and Atlanta, Georgia areas. The most intense areas remained Houston and Dallas.

Overall, the maps may suggest small area variations in practice patterns [27]. Although California and Florida have large populations, none of their major population centers reached the high-intensity scale of major cities in Texas. Furthermore, the Eastern seaboard’s increasing intensity suggests that something has changed. The questions then become are these changes in demand forecastable and how might they be explained.

### Forecasting Results

#### Number of Claims

Being able to forecast demand is necessary for decision makers to investigate both supply- and demand-side interventions. To that end, HTS for state, census bureau region, and the nation using both ETS and ARIMA components were built on 2012-2015 training dataset and compared with the 2016-2017 test set using the *hts* package in R [16]. Bottom-up, top-down, middle-out, and combination approaches to this forecasting were analyzed.

The ETS models performed better on the test set in terms of both variance and bias as shown (Table 6), and the middle-out model performed better on all bias (mean error and mean percentage error) as well as variance (root mean squared error, mean absolute error, and mean absolute percentage error) metrics. The overall forecast from the ETS middle-out model for the unobserved years {2016, 2017} was {454,720.3, 482,049.9}, whereas the actual overall claims were {464,323, 497,325}, resulting in mean absolute percent error (MAPE) of {2.0%, 3.1%}. Table 7 illustrates the forecast and actual number of claims at the region-level hierarchy for the best performing model, whereas Table 8 provides the state-by-state forecasts.

**Table 6:** The performance metrics of the various hierarchical models show that the exponential, trend, seasonality middle-out model performed best on the test set.

Model	Mean error	Root mean squared error	Mean absolute error	Mean percent error (%)	Mean absolute percent error (%)
ETS^a^-bottom up	860.42	2905.64	1371.81	3.16	10.71
ETS-top down	688.26	2423.58	1266.13	3.03	10.54
ETS-middle out	611.75	2256.70	1219.59	2.70	10.50
ETS-combination	682.27	2404.41	1235.04	2.25	9.56
ARIMA^b^-bottom up	5732.63	16496.72	5762.20	24.93	26.65
ARIMA-top down	5214.61	14953.59	5312.37	23.26	25.66
ARIMA-middle out	4606.38	13420.78	4799.88	20.70	24.20
ARIMA-combination	5159.04	14782.37	5259.67	20.65	25.92

^a^ETS: exponential, trend, seasonality.

^b^ARIMA: autoregressive integrated moving average.

**Table 7:** Region-level forecasts demonstrate small error. The average mean absolute percent error (MAPE) for {2016, 2017} was {3.4%, 6.2%}, respectively.

Region	2016 forecast	2016 actual	2017 forecast	2017 actual	MAPE 2016 (%)	MAPE 2017 (%)
East North Central	67,019	67,672	70,630	72,720	1.0	2.9
East South Central	42,765	42,747	44,477	47,107	0.0	5.6
Middle Atlantic	35,044	37,532	38,309	42,860	6.6	10.6
Mountain	35,840	35,254	38,211	38,341	1.7	0.3
New England	17,763	19,476	17,763	20,733	8.8	14.3
Pacific	45,015	43,647	49,122	43,865	3.1	12.0
South Atlantic	94,650	96,093	100,266	103,546	1.5	3.2
West North Central	45,653	46,550	48,075	48,600	1.9	1.1
West South Central	70,972	75,352	75,197	79,553	5.8	5.5

**Table 8:** Forecasts produced by the exponential, trend, seasonality middle-out model by state for 2016 and 2017 have an average mean absolute percent error (MAPE) of 10.1% and 13.2%, respectively.

State	2016 forecast	2016 actual	2017 forecast	2017 actual	Mean absolute error 2016 (%)	Mean absolute error 2017 (%)
Alaska	773	1,132	825	784	31.7	5.2
Alabama	13,709	14,029	14,658	15,697	2.3	6.6
Arkansas	6550	6193	7077	6150	5.8	15.1
Arizona	8450	7585	9438	9624	11.4	1.9
California	22,060	21,402	24,242	21,859	3.1	10.9
Colorado	7089	6736	7081	6512	5.2	8.7
Connecticut	2539	3103	2446	3289	18.2	25.6
Dist. of Columbia	1052	1015	1070	1041	3.6	2.8
Delaware	702	1129	683	1286	37.8	46.9
Florida	24,991	23,611	26,870	25,417	5.8	5.7
Georgia	15,324	14,293	16,372	14,093	7.2	16.2
Hawaii	369	451	370	527	18.2	29.8
Iowa	5613	5698	5624	6069	1.5	7.3
Idaho	3775	4755	3920	4793	20.6	18.2
Illinois	15,590	15,793	16,474	15,168	1.3	8.6
Indiana	14,839	14,839	14,850	14,648	0.0	1.4
Kansas	6943	7792	7382	7969	10.9	7.4
Kentucky	8061	7805	8044	9239	3.3	12.9
Louisiana	10,682	9817	11,519	11,044	8.8	4.3
Massachusetts	7251	8424	7533	9556	13.9	21.2
Maryland	6196	6458	6475	6785	4.1	4.6
Maine	3532	3707	3402	3345	4.7	1.7
Michigan	13,843	12,998	15,005	14,914	6.5	0.6
Minnesota	10,768	9,499	12,317	9507	13.4	29.6
Missouri	13,157	14,155	13,181	15,378	7.1	14.3
Mississippi	6943	7562	7252	7301	8.2	0.7
Montana	2954	2638	3116	2108	12.0	47.8
North Carolina	21,173	22,429	21,527	24,314	5.6	11.5
North Dakota	1839	1672	2017	2013	10.0	0.2
Nebraska	4437	4036	4576	4210	9.9	8.7
New Hampshire	3000	3073	2890	3382	2.4	14.5
New Jersey	6643	7067	7102	6659	6.0	6.7
New Mexico	1351	1570	1350	1755	13.9	23.1
Nevada	5067	4497	5606	5320	12.7	5.4
New York	12,876	14,206	14,014	15,442	9.4	9.2
Ohio	15,944	16,977	17,075	19,643	6.1	13.1
Oklahoma	9613	10,214	10,421	10,386	5.9	0.3
Oregon	7875	7,111	8454	7574	10.7	11.6
Pennsylvania	15,524	16,259	17,193	20,759	4.5	17.2
Rhode Island	841	695	914	750	21.0	21.9
South Carolina	11,050	10,921	11,858	13,494	1.2	12.1
South Dakota	2896	3698	2977	3454	21.7	13.8
Tennessee	14,052	13,351	14,523	14,870	5.3	2.3
Texas	44,127	49,128	46,181	51,973	10.2	11.1
Utah	6220	6077	6765	6811	2.4	0.7
Virginia	10,543	12,143	11,731	12,020	13.2	2.4
Vermont	600	474	578	411	26.6	40.6
Washington	13,937	13,551	15,231	13,121	2.8	16.1
Wisconsin	6803	7065	7227	8347	3.7	13.
West Virginia	3619	4094	3680	5096	11.6	27.8
Wyoming	935	1396	934	1418	33.0	34.1

**Figure 7:**
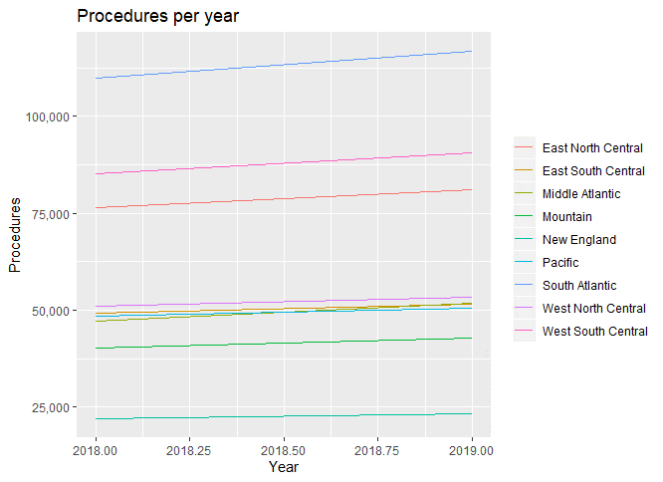
Regional forecasts generated by the hierarchical time series middle-out model with exponential, trend, seasonality components.

HTS with the middle-out approach and ETS methods was refit on the entire dataset to generate forecasts. Figure 7 shows the regional forecasts for 2018 and 2019. The East North Central region of the country is likely to experience the largest growth in claims. The overall demand for 2018 and 2019 is forecasted to be {529,777, 562,023}, which represents growth of 6.52% growth in the first year (from 497,325 procedures in 2017 to 529,777 in 2018) and 13.00% by 2019 (from 497,325 procedures in 2017 to 562,023 in 2019). At US $5000 average per claim (a simple linear model would suggest US $4910 in 2018 and US $5123 in 2019), the net increase in cost for 2018 would be US $162.2 million for 2018 and US $323.9 million for 2019. The next question becomes what explains the predicted growth of these claims other than possibly practice variation.

### Explanatory Modeling Results

To investigate explanatory variables, several models were explored. Stepwise regression for the number of claims at the hospital level using the transformed variables and an 80% training set was successfully able to predict the number of claims on the withheld test set with some accuracy (adjusted *R*^2^=0.39 on the training set and adjusted *R*^2^=0.38 on the test set). This indicates that the sum of squared regression accounted for 38% of the variance of the sum of squared total on the test set. Payments and charges were excluded from the analysis as they are necessarily functions of claims. The variables evaluated were the number of staffed beds, discharges, surgeries, net patient revenue, net income, total assets, total liabilities, affiliated physicians, employees, percentage Medicare/Medicaid, state, year, urban/rural status, ownership, medical school status, and hospital type. Table 9 provides the remaining variables generated from the stepwise regression at the hospital unit of analysis. It should be noted that obesity did not remain in the final model.

Stepwise regression for the number of claims with data aggregated (mean) by state and by year (N=306 observations, 51 states/territories × 6 years) resulted in an impressive model using an 80% training set to predict a 20% withhold set. The adjusted *R*^2^ was 0.87 on the training set and 0.77 on the test set after dropping insignificant variables from the analysis. The variables in this model included state, year, number of discharges, and total liabilities (a parsimonious model; Table 10). Again, there is no evidence that obesity rates are predictive of CPT 63 surgery in this model.

Lasso, ridge, and elastic net regression models were able to predict the unaggregated test set with some accuracy (*R*^2^=0.38, 0.37, 0.38, respectively.) None of these penalty-weighted models improved upon the stepwise analysis significantly, although elastic net tied. Obesity was not retained in these models. For the aggregated set (state and year), the associated *R*^2^ were 0.78, 0.75, and 0.78, respectively. The lasso and elastic net models were slightly superior to the stepwise regression model (Figure 5). The top 10 variables by effect size in the state-aggregated elastic net model are shown in Table 11. The effect size of obesity was near zero (0.0098). If one were to make a conclusion using traditional and constrained linear models, obesity would not be a factor for explaining the number of claims; however, random forests would prove otherwise.

**Table 9:** Variables below from the stepwise regression predicted a withhold set with adjusted *R*^2^=0.38.

Variable	Sum of squares	Mean squared error	*F* value (*df*)	*P* value
Staffed beds	95.78	95.78	2423.29 (1)	<.001
Discharges	21.93	21.93	554.77 (1)	<.001
ER visits	17.50	17.50	442.68 (1)	<.001
Surgeries	41.15	41.15	1041.03 (1)	<.001
Net patient revenue	5.57	5.57	140.99 (1)	<.001
Net income	2.53	2.53	63.97 (1)	<.001
Total liabilities	4.45	4.45	112.70 (1)	<.001
Affiliated physicians	0.24	0.24	6.08 (1)	<.01
Employees	6.28	6.28	158.78 (1)	<.001
Percentage Medicare/Medicaid	1.16	1.16	29.34 (1)	<.001
State	52.35	1.05	26.49 (50)	<.001
Year	8.09	1.62	40.91 (5)	<.001
Urban rural status	1.99	1.99	50.43 (1)	<.001
Ownership	10.76	0.90	22.68 (12)	<.001
Medical school affiliation	2.16	0.54	13.63 (4)	<.001
Hospital type	0.64	0.13	3.24 (5)	<.01

**Table 10:** Variables in the analysis by state and by year.

Variable	Sum of squares	Mean squared error	*F* value (*df*)	*P* value
State	1.16	0.02	26.40 (50)	<.01
Year	0.17	0.03	39.16 (5)	<.01
Discharges	0.06	0.06	63.19 (1)	<.01
Net income	0.004	0.004	4.45 (1)	.04
Total liabilities	0.004	0.004	4.65 (1)	.03

**Table 11:** Top 10 coefficients by effect size of the elastic net.

Variable	Coefficient
Total assets	−1.539
Net patient revenue	−1.044
Number of staffed beds	0.212
Number of discharges	0.186
New Jersey	−0.178
Total surgeries	0.162
New York	−0.159
California	−0.145
Delaware	−0.143
Employees	−0.136

**Figure 8:**
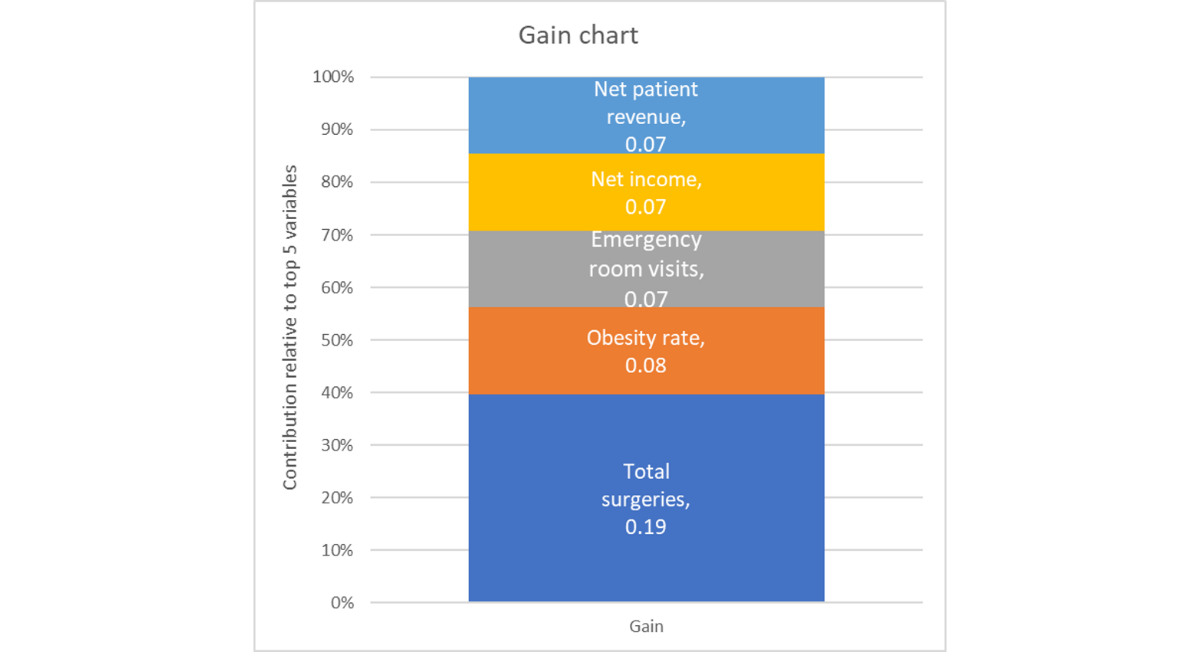
Gain plot for the top 5 variables, unaggregated model.

**Figure 9:**
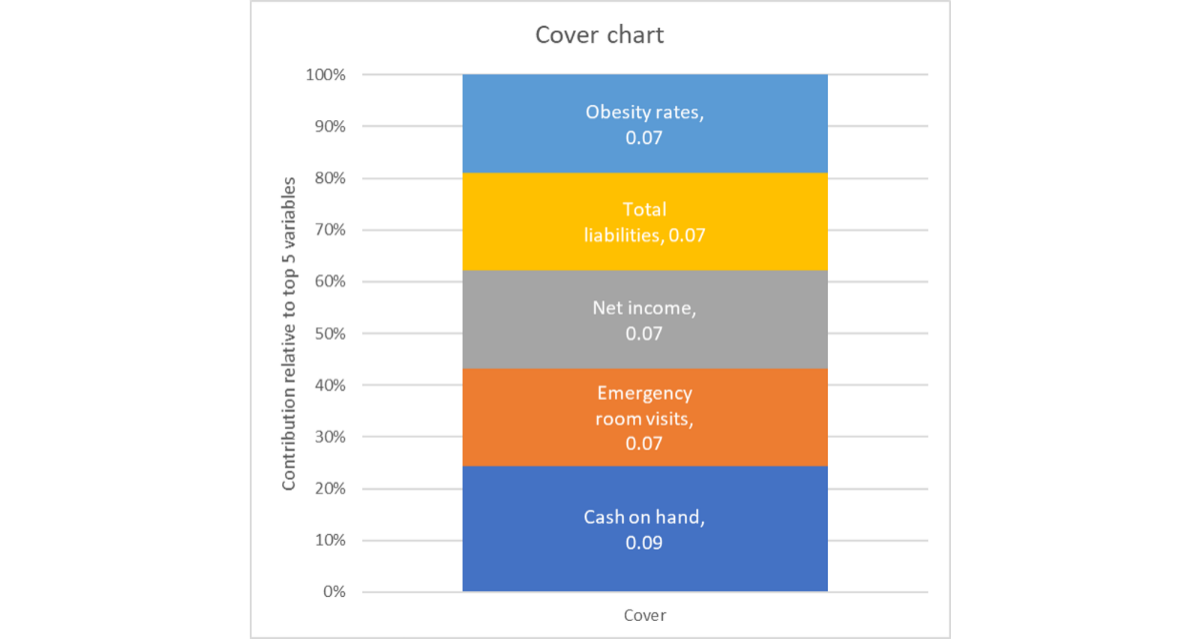
Cover plot for the top 5 variables, unaggregated model.

Gradient-boosted random forests with hyperparameter tuning outperformed all models: stepwise, lasso, ridge, elastic net regression. On the unaggregated withhold set, a well-pruned model (depth 4) with 2000 epoch runs and a slow learning rate of 0.1 accounted for more than 78.5% of the variability (*R*^2^=0.79) on the unobserved test set. Comparing this value with the approximately 38% variability accounted for in the other models suggests that the random forest model is superior. Figure 8 is a plot of the gain (the average improvement when the feature is used in a tree) for the top 5 items in the importance matrix, whereas Figure 9 is a plot of the cover (the average proportion of samples affected by splitting using this feature) for the top 5 items of the unaggregated model. These figures illustrate that obesity is one of the prominent features in both gain and cover of the unaggregated model.

**Figure 10:**
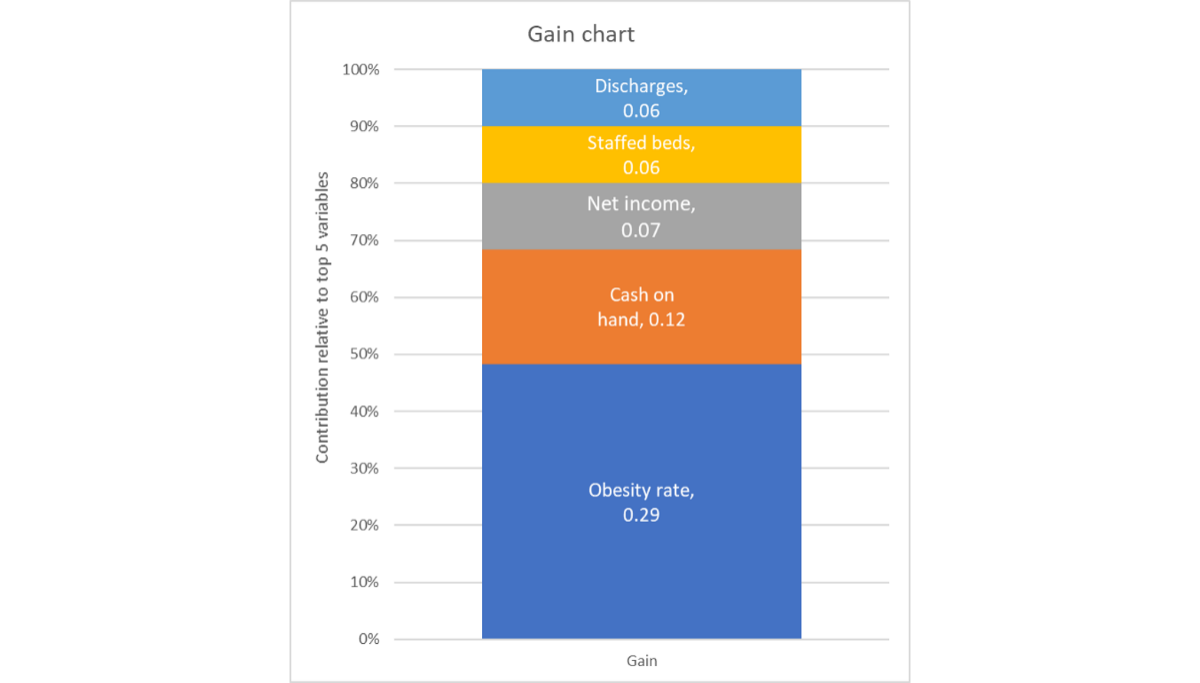
Gain plot for the top 5 variables, aggregated model.

**Figure 11:**
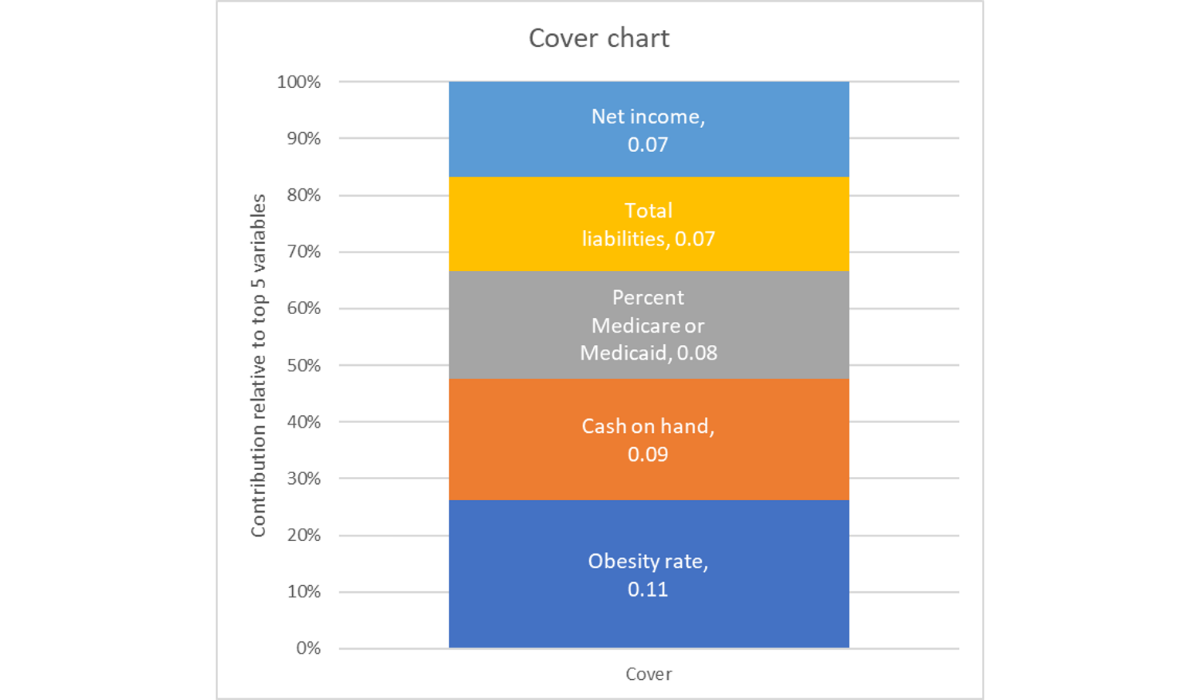
Cover plot for the top 5 variables, aggregated model.

Despite the exceptional gains of the extreme gradient-boosted random forests on the unaggregated, hospital-level data, the application of hyperparameter-tuned models to the aggregated data (by state and year) yielded only nominal improvement over the constrained regression methods, possibly because of the smaller sample due to aggregation. A well-pruned model (depth=3) after 3000 epochs with a slow learning rate (0.1) achieved an *R*^2^ of 0.80. The gain and cover graphs are shown in Figures 10 and 11, and obesity rate is the most important feature at the state-aggregated level.

Most importantly, the gradient-boosted random forests identified obesity as the second most important factor for gain at the hospital level and as the most important factor for both gain and cover at the state level of analysis. Furthermore, the gradient-boosted random forests performed better than any other model considered on a blinded test set.

## Discussion

### Principal Findings

In this analysis, we evaluated the location, magnitude, and reasons for the growth of CPT 63 back surgeries in the United States. The GIS heat map analysis shows large-scale growth, particularly in the Northeastern region of the United States, and sustained activity in Texas. The entirety of the Eastern seaboard has seen growth in these procedures, and the associated increased cost is estimated to be US $323.9 million by the end of 2019.

The principal findings of this study are described here. Each of the following results includes a discussion of significance and (if appropriate) policy:

1. The Northeastern seaboard is likely to see continued growth in CPT 63 procedures. The implication for states in this region is that they may see more unplanned expenditures on health care, affecting their budgets. Furthermore, cost controls and reduction of practice variation based on evidence will become more important.
2. The cost associated with these procedures is outstripping inflation and will likely result in national expenditures in the triple-digit billions. The federal government may need to evaluate its own evidence-based, best practice policies associated with funding of procedures that link selected interventions with outcomes and that reasonably limit reimbursement.
3. Interstate practice variation appears to be extreme. For example, large population centers in California have fewer claims than large population centers in Texas. States should also investigate intrastate variation.
4. Hierarchical forecasts suggest an increase in the number of claims of 6.5% for 2018 and 13% in 2019. The initial models were built on a blind test set and performed well. These types of forecasts are reasonably effective for claims analysis.
5. Explanatory regression models for nation-level claims data had only some success in internal predictions. These models excluded obesity as a predictor. Regression models were more successful at predicting aggregated state/year models, though. These traditional models should be abandoned in favor of random forests.
6. Extreme gradient-boosted random forest models were highly successful in predicting both hospital-level unit of analysis number of claims and aggregate-level claims on an unobserved test set. These models identified obesity as an important factor in estimating the number of claims. Furthermore, the use of these models underscores that even after multivariate transformations, nonlinear functions may exist in modeled data. Random forests unearthed patterns not visible to regression and constrained regression models.

### Limitations

There are many limitations in this work. First, the algorithms used by Definitive Healthcare to extrapolate CMS data to *all-payor* data are not divulged. This omission is problematic for verification but understandable because of parochial concerns. Second, only ETS and ARIMA models were considered for the HTS fitting as these models are implemented in the R HTS package. There are an infinite number of models for forecasting, including random forest time series that might have performed better. Third, the explanatory variables are limited to those tracked by CMS and the BRFSS.

### Conclusions

Hospital-based back surgeries are likely to increase dramatically over the next several years, yet the supply of neurosurgeons is constant. With that increase, the cost of the procedures (mostly borne by third-party payers) will increase as well. Practice variation appears to be prevalent across the country; however, obesity itself is a factor that must be considered as a significant influence. Policy interventions must be considered at many levels.

Clinical practice variation is something that may require intervention at the federal level. For example, a study in Scandinavia found significant differences among Norway, Sweden, and Denmark in the use of concomitant arthrodesis without any difference in treatment efficacy, increasing the cost without improving outcomes [28]. Controlling costs across states may require federal (and state) reimbursement interventions and incentives.

States should continue educational and financial interventions targeting obesity in adults and children. As the obesity epidemic continues to grow, the medical intervention costs are likely to grow accordingly. Furthermore, states should evaluate county-to-county practice variation as these variations often increase cost without improving quality [27].

Local interventions should consider the targeting of food deserts (urban areas where fresh, quality food is difficult to find) for eradication as well as educational interventions. Several studies have shown that the food environment is directly linked to obesity [29-31]. Eliminating or at least reducing the number of food deserts requires incentivizing grocery stores to populate areas where it may not be as lucrative because of poverty or demand.

Insurance companies themselves have a vested interest in both reducing obesity and controlling practice variation. Obesity is linked to numerous health disorders such as heart disease, type 2 diabetes, and bone and joint disease [32], any of which may result in additional costs to the health care system and insurer. Funding prevention efforts and establishing policies to reduce practice area variation are likely to benefit them as well as the population health over time.

Federal, state, and local policy makers need to address the increasing obesity epidemic and the likely associated increase in demand for back surgeries. The implications of not doing so are increased cost, questionable quality/cost trade-offs, and reduced access because of the small and steady number of available neurosurgeons. The *fattening of America* and the costs associated with it are likely to continue increasing otherwise.

## Appendix

Multimedia Appendix 1 This video depicts heat maps for the number of claims from 2012 through 2017.

## Abbreviations

ARIMA: autoregressive integrated moving average models
BRFSS: Behavioral Risk Factor Surveillance System
CPT: Current Procedural Terminology
CMS: Centers for Medicare and Medicaid Services
ETS: exponential, trend, seasonality
HTS: hierarchical time series
MAPE: mean absolute percent error
